# Substance Use among Youth in Community and Residential Mental Health Care Facilities in Ontario, Canada

**DOI:** 10.3390/ijerph19031731

**Published:** 2022-02-02

**Authors:** Oluwakemi Olanike Aderibigbe, Shannon L. Stewart, John P. Hirdes, Christopher Perlman

**Affiliations:** 1 School of Public Health Sciences, University of Waterloo, Waterloo, ON N2L 3G1, Canada; hirdes@uwaterloo.ca (J.P.H.); chris.perlman@uwaterloo.ca (C.P.); 2 School and Applied Child Psychology, Faculty of Education, University of Western Ontario, London, ON N6G 1G7, Canada; sstewa24@uwo.ca

**Keywords:** substance use, youth, mental health, biopsychosocial, interRAI, assessment

## Abstract

There is a need to improve the integration of substance use and mental health care for children and youth. This study examines risk and protective factors for substance use among youth with mental health conditions who received community-based or residential care services between 2012–2020 in Ontario, Canada. In this study, a cross-sectional design was used to examine patterns and factors associated with substance use among youth (12–18 years) assessed in the community (n = 47,418) and residential (n = 700) mental health care facilities in Ontario, Canada. Youth were assessed with the interRAI Child and Youth Mental Health Assessment (ChYMH). Substance use is identified by any substance use (including alcohol) 14 to 30 days prior to assessment. Logistic regression with generalized estimating equations was used to examine clinical, psychosocial, and environmental factors associated with substance use. This study shows that 22.3% of youth reported the use of substances in the community settings and 37% in residential settings. Older age group (Youth older than 16 years), being a victim of abuse, having experienced self-injurious ideation/attempt, being at risk of disrupted education, and having a parent/caregiver with addiction or substance use disorder were significantly associated with substance use. Several factors reduced the risk of substance use, including being a female, having anxiety symptoms, and having cognition problems. In conclusion, the study found that individual and parental factors increase youth’s risk of substance use, highlighting the importance of a holistic approach that includes consideration of social and biological risk factors to prevention/risk reduction, risk assessment, management, and recovery.

## 1. Introduction

Substance use disorders are one of the three most common types of mental illnesses experienced by Canadians, with young people aged 15–24 years having the highest rates of substance abuse or dependence [1]. Initiation of substance use may include experimentation or heavier and higher risk patterns of use [2,3,4]. Alcohol has been the most commonly used substance among youth, followed by tobacco, cannabis, and opioids [5,6]. School surveys in Canada have reported that up to 60% of students in grades 7–9 reported lifetime use of various substances [7,8]. In Ontario, Canada, 75% of youth in grade 12 reported lifetime alcohol use, 26% cannabis, and 26% nicotine [6]. Another study that examined current users of substances (use of substances in the last 30 days) shows that among youth in grades 7 to 12 in a general Canadian population sample, 27% reported current use of alcohol, 19% reported cannabis use, and 8% reported other drugs [9]. Substance use among youth can have a cumulative impact, contributing to costly social, physical, and mental health problems [2,3,4,10,11]. Substance use conditions are associated with problems such as poor academic performance, job instability, teen pregnancy, the transmission of sexually transmitted diseases, accidents, crimes, violence, overdoses, drug tolerance effects, withdrawal symptoms, and other longer-term physical health issues [11,12,13]. Other problems associated with problematic substance use include loss of interest in social activities, disorganized thinking, reduced problem-solving performance, and social isolation [2,3,4,10].

The biopsychosocial model provides a comprehensive explanation of the complex interactions that can occur leading to substance use. This model explains that biological, genetic, personality, psychological, cognitive, social, cultural, and environmental factors interact to produce substance use disorder [14]. Genetic and biological risks include being children of alcohol-dependent parents or the presence of the alcohol dehydrogenase enzyme and dopamine D2A1 gene. For instance, a study examining the association between the gene responsible for alcohol dehydrogenase and alcohol use among the Asian population found that those deficient in this enzyme are less likely to use the substance [15]. However, not all persons with such predispositions develop problems with substance use. A myriad of other psychosocial factors may promote the use of substances, including individual, familial, social, and environmental factors [16]. Factors at the individual level include being a male, LGBTQ+, early exposure to traumatic life events, individuals with a family history of a substance use disorder, prenatal exposure to alcohol and other drugs, sleep problems, and co-occurring psychopathology (e.g., ADHD, depression) [2,3,17,18]. Social factors include peer substance use and involvement in romantic relationships at an early age [19,20]. Environmental risks include family dysfunction, lack of parental supervision and monitoring, and being street-involved [2,3,4,10,11]. There are also protective factors, including self-efficacy, social competence, academic achievement, personal and social controls against norm-violating behaviors, church attendance, and sense of morality [21,22,23,24,25,26]. Nurturing home environments with open communication and parental support as well as positive school experience and positive peer role models may also be protective [23,24].

Substance use during a young age might disrupt vital developmental transitions as the brain undergoes cognitive, emotional, and psychosocial development [1]. In addition, this period also coincides with the onset of a variety of mental health conditions [27,28,29,30]. Neurodevelopmental research has demonstrated that the brains of youth may be more vulnerable to the effects of substances. Hence, they are at risk of developing patterns of behavior that result in substance abuse (continued use regardless of physical or psychosocial problems or dependence) and substance dependence (physiologic dependence demonstrated by withdrawal symptoms or the development of tolerance to alcohol or other drugs) [14,31,32]. These adverse outcomes are particularly concerning for youth who use substances in an attempt to cope with difficult situations such as trauma or victimization due to the dual neurological vulnerability created by traumatic life events and the engagement in substance use [20,23,33,34,35,36,37].

There is a consistent relationship between mental health conditions and problematic substance use among youth [16,17,18,23,24]. Co-occurring mental and substance use conditions are highest among youth aged 15–24 years in Ontario and Canada [2]. Substance use is associated with comorbid illnesses such as depression and bipolar disorders, post-traumatic stress disorder, psychotic symptoms, and disruptive behavioral disorders in both in-patient and outpatient settings [26,38,39]. For youth with mental health conditions, early initiation of treatment is significantly associated with a reduced likelihood of developing substance use disorders [26].

Mental health and substance-related service use among young people in Ontario, Canada, has increased over time, with about 1 in 6 receiving needed mental health services [40]. Co-occurrence of substance use also interferes with the treatment of and recovery from mental health disorders, thus requiring specific consideration within the treatment plan and the need for integrated health services [41,42]. Although best practice promotes the integration of mental health and substance use services, evidence suggests this is rarely the case [43]. In Ontario, some historical barriers to integration have included variation in the public funding and administration of youth mental health services and substance use care, as well as variations in the eligibility criteria for services, particularly related to the presence of substance use [43,44,45]. Consequently, youth have experienced challenges accessing and navigating services across multiple service sectors in Ontario [44,45,46,47]. There are also substantial delays between the onset of mental health or substance use conditions and access to care, with only the severe cases receiving timely services [48,49]. While recent amendments to public policy have been made to the public administration of child and youth mental health services in Ontario [50], gaps often remain in the integration of mental health and substance use care at the provider level. Youth seen in outpatient settings and those admitted into residential/in-patient settings may demonstrate greater clinical complexity and/or higher rates of substance use compared to youth in the general population.

There is a need to improve the early identification of comorbid mental health conditions and modifiable risk factors associated with substance use. This will help to develop early intervention strategies to potentially reduce the risk of developing substance use disorders and the utilization of high-intensity services. Therefore, this study examines individual factors associated with substance use among youth (12–18 years) accessing services in community-based and residential mental healthcare settings in Ontario, Canada.

## 2. Materials and Methods

### 2.1. Methodology

#### 2.1.1. Study Population

The sample comprised 48,118 youth (12–18 years) assessed in the community (N = 47,418) or residential (N = 700) mental health agencies in Ontario, Canada, with either the interRAI Child Youth Mental Health Assessment (interRAI ChYMH) or the interRAI ChYMH Screener (ChYMH-S) between 1 January 2012 data, and 31 October 2020. Referrals were made to the agencies through family physicians, pediatricians, school personnel, parents, or other allied professionals. Data from the first assessment of all youth were selected for the analyses. As part of the license to use the interRAI ChYMH data, sharing agreements are in place for agencies to submit anonymized data to interRAI Canada at the University of Waterloo.

#### 2.1.2. Instruments

The interRAI ChYMH is used as part of the standard of care in many agencies and organizations that provide service to children and youth across Ontario. The assessment includes over 400 items to inform treatment planning, including identification information, intake and initial history, mental state indicators, substance use or excessive behavior, harm to self and others, behavior, strengths and resilience, cognition and executive functioning, independence in daily activities, communication and vision, health conditions, family and social relations, stress and trauma, medications, preventions, service utilization and treatments, nutritional status, education, environmental assessment, diagnostic and other health information, service termination and discharge information. Items in the assessment are completed by trained clinicians overseeing the care of the person. Assessors use a semi-structured interview approach that incorporates all available information, including observation, review of the clinical record, and parent report as well as self-report [51]. The ChYMH also includes the interRAI Adolescent Supplement Assessment Form [49]. This supplement to the ChYMH is used when assessing youth aged 13 to 17. It includes additional information on substance use or excessive behavior, independence in daily activities, service utilization, and strengths. The interRAI-ChYMH has demonstrated good psychometric properties in clinical settings [52].

The ChYMH-S is a brief screener that provides an initial assessment for early identification, triaging, and prioritizing services. Taking approximately 15–20 min to complete, the assessment includes the following domains: (1) Mental State Indicators (e.g., Mood disturbance, Anxiety), (2) Substance Use or Excessive Behaviour, (3) Harm to Self and Others, (4) Behaviour, (5) Cognition, Communication and Development, (6) Stress, Trauma, and Social Relationships, and (7) Education. The screener demonstrated strong inter-item reliability on all measured scales and good convergent validity [53].

Dependent Variable: The primary variable of interest was the use of any substance, including misuse of medication and alcohol, in the 14 to 30 days prior to assessment. The ChYMH Adolescent Supplement assesses the most recent instance a youth reported using a given substance, including opiates, cannabis, cocaine, stimulants, inhalants, hallucinogens, and intentional misuse of prescription or over-the-counter medication. Use is coded if the person used the substance in the three days, seven days, 30 days, one year, and more than one year before assessment. The ChYMH-S assesses whether the person used substances or intentionally misused prescription or over-the-counter medication in the 14 days before the assessment.

Independent Variables: The interRAI ChYMH and ChYMH-S include a range of clinical, functional, social, and familial variables that were considered in the biopsychosocial model in the examination of factors associated with youth substance use. The biopsychosocial model posits that biological, genetic, personality, psychological, cognitive, social, cultural, and environmental factors interact, leading to the use of substances and substance use disorders [14]. Using this model as a framework, variables in the ChYMH and ChYMH-S could be grouped into demographic factors that include age, sex, education, employment, and insurance coverage. Cultural factors included aboriginal identity and if an interpreter was needed to complete the assessment. Clinical characteristics included psychological, personality, and cognitive factors, and psychosocial categories included developmental needs, housing, income and family-related variables, community, and education engagements (See Table 1 and Table A1). However, the psychosocial model of adolescent substance use was preferred because it contains modifiable factors that could be addressed. Therefore, variables for the final model were modifiable factors that are available interRAI assessment instruments.

In addition to these items, this study examined three scales embedded in the ChYMH and the ChYMH-S: Anxiety Scale, Distraction/Hyperactive Scale, and Depression Severity Index. The Anxiety Scale is a seven-item scale that assesses the frequency of several anxiety symptoms (i.e., repetitive anxious concerns, unrealistic fears, obsessive thoughts, compulsive behavior, intrusive thoughts or flashbacks, episodes of panic, and nightmares). The total sum of the frequency of each symptom (4-point scale where 0  =  not present to 4  =  Exhibited daily in last 3 days, 3 or more episodes or continuously) ranges from 0 to 28. Thus, higher scores indicate higher levels of anxiety [52]. The Cronbach’s alpha for the Anxiety Scale was 0.75 in the current sample. Hyperactive/Distraction Scale is a 4-item scale that assesses the frequency of four facets of distractibility and hyperactivity. That is impulsivity, ease of distraction, hyperactivity, and disorganization). The total sum of the frequency of each behavior (0  =  not present to 4  =  Exhibited daily in the last 3 days, 3 or more episodes or continuously) ranges from 0 to 16. Higher scores indicated higher levels of distractibility and hyperactivity [52]. The Cronbach’s alpha for the Hyperactive/Distraction scale was 0.77 in the current sample. The Depression Severity Scale is comprised of five items, capturing various depressive expressions of the individual, including sad or pained facial expressions, made negative statements, self-deprecation, expressions of guilt/shame, and hopelessness. Possible scores for each of the items are 0 (“Not present”), 1 (“Present but not exhibited in last 3 days”), 2 (“Exhibited on 1–2 of last 3 days”), and 3 (“Exhibited daily in last 3 days”). Higher scores indicated higher levels of depressive symptoms [54]. The Cronbach’s alpha for the Depression Severity scale was 0.83 in the current sample. The Cronbach’s alphas for the three scales in this study sample were more than 0.7, indicating good internal consistencies.

#### 2.1.3. Analytic Approach

Separate analyses were done for the residential and community datasets. Descriptive analysis of the study population performed includes frequency and percentages for categorical data for both settings. Bivariable relationships between the independent variables and substance use were examined using chi-square analyses due to the categorical nature of the data.

Modified Poisson GEE regression model was used to estimate the effect size of the association between the independent variables and substance use for variables with significant bivariable associations to estimate the relative risk for community and residential datasets. Regression models using GEE are considered marginal, or population-averaged, models that account for clustering of observations (i.e., correlation of responses) within organizations by including organization identification number (ID) as a source of random error in each model [55,56,57]. The GENMOD Procedure in the Statistical Analysis Software version 9.4 was used to assess the risk ratio with a 95% confidence interval using a REPEAT statement to specify the GEE procedure. The organization ID in the ChYMH dataset was entered as the clustering variable using the Subject option (subject identifier). The independent correlation structure assumes that each individual and individual subject is independent [58].

The variables were entered in blocks into the model (e.g., biological factors, psychosocial factors, environmental factors, and clinical factors). Different combinations of the independent variables were examined to rule out order-of-entry, deletion effects, and multicollinearity [58,59]. For inclusion in the final risk model, variables needed to be statistically related to substance use (i.e., parameter estimates with *p*-values less than 0.01) with risk ratios greater than 1.3 or below 0.77.

Many methods to evaluate the goodness of fit for GEE regression models have been proposed in simulation studies [60]. For instance, the goodness of fit statistics QIC and QICu can compare the models’ strengths with lower QIC and QICu values indicating better model performance [61]. However, these methods are not routinely implemented in SAS output, and consensus on the goodness of fit statistic for GEE models has not been established. Therefore, final risk models using GEEs identified in the ChYMH dataset were subjected to logistic regression. Using logistic regression, the model’s discriminatory power was evaluated using the c statistic [62]. The c statistic measures how well the model discriminates those who experience an event (e.g., outcome) from those who do not [63]. For example, a c statistic of 0.5 indicates the model is no more discriminating than chance, while a statistic of 1.0 indicates perfect discriminatory power.

## 3. Results

Descriptive and clinical characteristics of the community and residential sample are in Table 2. The community sample was predominantly female (60.4%), while just over half of the residential sample were male (53.9%). About a third of youth in the community sample were victims of physical, sexual, or emotional abuse (36.4%) compared to 59.0% within the residential sample. The majority of youth assessed in the residential and community settings had contacts with community mental health agencies or professionals prior to assessment, 87% and 69%, respectively.

There were several differences in clinical characteristics among males and females in the community agencies. For instance, a larger proportion of females reported self-injurious ideation (67.7%), anxiety (87.1%), and abuse (41.0%), compared to males (43.7%, 76.1%, and 29.3%, respectively). Alternatively, a higher proportion of males experienced behavioral symptoms (62.9%) and cognitive problems (23.6%) compared to females (49.6% and 13.4%, respectively). In the residential settings, a higher proportion of females reported a lack of strong family support (40.6%), having a parent with MH issues (53.9%), history of self-injurious ideation/attempt (79.6%), abuse (63.5%) and anxiety (81.1%) compared to males (28.4%, 46.4%, 6.4%, 53.3%, 55.2%, and 73.7% respectively). More males experienced cognitive problems (54.9%), and HDS (91.0%) compared to females (26.0%, 36.8%, and 85.2%, respectively). The proportion of youth who reported any substance use in the 14–30 days before the assessment was larger in the residential settings (37.1% females and 36.9% males) than in the community setting (22.1.0% females and 22.5% males).

Bivariable analyses of factors associated with substance use in the 14–30 days prior to assessment for the community sample are shown in Table 3 and Table 4 for the residential sample. While most variables had a significant bivariable association in community settings, variables associated with substance use in residential settings were limited to age, self-injurious ideation/attempt, behavioral symptoms, cognitive problem, being a victim of abuse, being at risk of disrupted education, lack of family support, and having parent/caregiver with SUD. There was no statistically significant difference in the proportion of males compared to females that have used substances in the last 14–30 days in the community (bivariate OR = 1.02, 95% CI: [0.98, 1.07]; *p*-value = 0.31) and residential (bivariable OR = 1.01, 95% CI: [0.74, 1.38]; *p*-value = 0.94) settings. However, the proportion of youth who used substances significantly increased as youth age increased. In the community setting, the proportion of youth who used substances increased with age from 5.0% of youth aged 12–14 to 36.8% of youth aged 17–18. In the residential setting, the proportion of youth who used substances increased from 16.0% among youth aged 12–14 to 49.6% of youth aged 17–18. This pattern of substance use across age categories was similar among males and females in both community and residential settings. However, the proportion increased more by age for females compared to males.

### Factors Associated with Substance Use among Youth Assessed in Outpatient and In-Patient Mental Health Agencies in Ontario

Table 5 shows the results of the GEE models showing factors associated with substance use among the total samples and by gender in community and residential mental health settings. Only variables that were statistically significant are shown in the models. Among youth assessed in the community (N = 47,418), holding all other variables constant, the relative risk of substance use is significantly lower among females compared to males (RR = 0.87, 95% CI: [0.82, 0.93]). Contact with mental health agencies, sleep problems, DSI, and having parents/caregivers with mental health issues were not significantly associated with substance use in the community sample. Factors associated with a decreased relative risk of substance use include being female, having anxiety symptoms (ANX Scale), and cognition problems were associated with reduced risk of substance use in the community sample.

In residential/inpatient settings (N = 700), similar factors to those in the community sample were associated with substance use (See Table 5). However, several factors not associated in the residential setting include being female (RR = 0.91, 95% CI: [0.71, 1.16]), parent mental health issue (RR = 0.92; 95% CI: [0.76, 1.12]), no strong family relationship (RR = 1.11, 95% CI: [0.93, 1.33]), behavioral symptoms (RR = 1.33, 95% CI: [0.98, 1.82]), problematic sexual behavior (RR = 1.11, 95% CI: [0.75, 1.64]), sleep problem (RR = 1.15, 95% CI: [0.93, 1.42]), DSI, hyperactivity and distractibility symptoms and anxiety scales include 1:00 (no difference in risk).

## 4. Discussion

This study demonstrates the clinical complexity of youth who use substances and those who may require substance use treatment in the community and residential clinical settings across Ontario, Canada. Interestingly, the pattern of substance use among youth in community mental health setting is different than those in the general population. For instance, among youth in grades 7 to 12 in a general Canadian population sample, 27% reported current use (use in the last 30 days) of alcohol, 19% reported current cannabis use, and 8% reported other drugs [9], while this study shows that 11% of youth in the community mental health settings reported current alcohol use. Furthermore, the proportion of youth who reported use of illicit drugs is lower in the general population (8%) than community mental health (interRAI ChYMH) sample (17%), and 22% of youth in the community sample reported current use of any substance (including alcohol, cannabis, illicit drugs, and misuse of prescribed or over the counter medications). The nature of the items on the interRAI ChYMH-Screener, representing the majority of the sample, limited the ability to assess lifetime use of substances and alcohol. As might be expected, results from the residential sample confirmed that substance use is much more common among youth experiencing severe mental health concerns than those in the community sample in this study and general population samples from prior literature.

Understanding the reasons for substance use among youth can be challenging, as suggested by the biopsychosocial model of substance use and the wide range of psychosocial factors associated with substance use [14,16]. Although the results of this study are cross-sectional, the findings do offer insights into a range of circumstances that may be related to youth substance use. This study found that individual, familial, social, and environmental factors contribute to the risk of substance use among youth. Most factors associated with substance use were similar across clinical settings. This study found that several clinical variables that were statistically significant in the community were not significant for the residential sample. These included time since contact with CMHA, Hyperactivity/Distractibility symptoms, sleep problems, problematic sexual behavior, and having a parent with a mental health problem. However, we could not examine these differences extensively due to a lack of statistical power of the small sample size in the residential sample. Also, some of the differences may be due to the rarity of the clinical symptoms among children and youth (e.g., problematic sexual behavior). Furthermore, the difference may be related to the case-mix of youth admitted into residential care. For instance, barriers regarding access to care in the community may delay timely help-seeking resulting in complicated cases in need of urgent care or direct admission to residential care.

This study found that females (60.17% of the sample population) were more commonly assessed compared to males (39.83%) in the community settings, but more males (53.9%) than females (46.1%) in the residential settings. While there was no significant difference in the proportion of males and females who used any substance in community and residential settings, this study showed that females were more likely than males to report alcohol use and misuse of prescription or over-the-counter medication. In contrast, males were more likely than females to use illicit drugs. In the community sample for this study, the proportion of youth using substances was almost identical between males and females. However, after controlling for other variables, the relative risk of substance use was significantly lower among females compared to males. While the patterns of substance use were similar by sex, it is crucial to consider other unique factors related to substance use among youth of each sex. For example, we know that the brain, hormones, and metabolic systems differ between males and females, such as differences in body weight and metabolism of substances such as alcohol [64]. These differences may relate to the experiences and effects induced by drugs among each sex. For instance, the differences in the subjective effects (adverse or rewarding) experienced by males and females may affect the continued use of substances. Also, males are more likely than females to engage in risky behaviors, including substance use, in anticipation of perceived social rewards or peers’ influence. Other factors that could be influencing this association include parental factors like gender, caregiving capacity, and substance use or addiction. This finding is important to consider within clinical practice and public health. For instance, substance use screening and counseling could be mandatory for prevention and early interventions for boys.

This study shows that substance use was common, particularly among those of older age and in residential settings. While the pattern of substance use by age was similar for male and female youths in the community and residential settings, the proportion of substance use by age categories increased more for females than males in the residential settings. The relationship between age and substance use observed in this study was not surprising but underscored the potential utility of this study’s findings for early intervention. Previous studies have also shown a strong association between the onset of substance use and the development of substance use disorder later in life [65]. For instance, excessive and early initiation of substances like cannabis increases the risk of schizophrenia among young adults [65]. This association highlights the importance of early prevention efforts to reduce potential risky behaviors and long-term adverse health effects. The finding that a subset of youth aged 12–14 used substances shows that prevention and intervention should also target younger age groups (i.e., less than 12 years).

The finding that parental mental health status was related to substance use reinforces prior literature demonstrating strong associations between genetic/familial factors and substance use [66,67]. Therefore, early and comprehensive interventions among youth with a history of parental substance issues could prevent future use. For instance, children of alcohol-dependent parents were more likely to use a substance [68,69]. While children of parents deficient in alcohol dehydrogenase enzyme and dopamine D2A1 gene are less likely to use the substance. Environmental factors like parental substance use and permission of substance use in the home increase the likelihood of offspring substance use. Parents that use substances could be modeling this behavior to their offspring or permit them to use substances under their supervision. Parents with substance use disorder may also lack the capacity to supervise or adequately parent their children. Therefore, care planning for this population should be based on multidimensional approaches that focus on the importance of intervening across multiple levels of socialization, including parents. For instance, parents should be engaged in the care planning and treatment of youth at risk or with substance use issues.

The results of this study reinforce prior theories on the roles that psychosocial factors like family and school experience play in substance use among youth, emphasizing the importance of their engagement in the prevention of substance use among young people. Strong parental/family support was found to be a protective factor against substance use among youth in this study, as were positive school experience and academic achievement. This study shows that even youth who were not at risk of disrupted education because they were homeschooled were more likely to use substances than those in school and at risk of disrupted education. This finding shows the importance that school enrollment may play in substance use. Perhaps the school environment provides social structure and engagement and a network for monitoring youth health behaviors even in light of existing mental health concerns. There is a need for further research on this finding.

Strong clinical predictors of substance use among this study sample include Hyperactive/Distraction, having a history of self-injurious ideation/attempt, and exposure to or experienced traumatic life events (physical, sexual, and emotional abuse). Substance use may precede psychopathologies, as youth who use substances may engage in risky behavior that increases their risk of experiencing trauma leading to PTSD [70]. Psychopathology may precede youth substance use. For instance, youth with a behavioral or psychological problem may have impaired learning or impulsivity, leading to an inability to conceptualize the consequences of substance use. They may also be experiencing school failure leading to associations with deviant peers and self-medication. Also, the association between youth substance use and these clinical predictors may be due to shared vulnerability of genetic predisposition and childhood psychosocial factors. However, in contrast to some previous studies, anxiety symptoms and cognitive impairment were associated with reduced risk of substance use among male and female youths in the community sample after controlling for other factors.

## 5. Limitations

This study is cross-sectional; therefore, these results should be interpreted with care since it did not test temporality or establish causality. Also, study sample selection was biased since only those referred to service providers were assessed. Furthermore, this study examined a small sample size for the residential sample, limiting the confidence of this study’s conclusions about the findings. In addition, this study did not examine peer support which is another important factor considered in youth substance use. There is also no data on tobacco use or the types, dose, mode of administration, and frequency of substance use. In addition, this study did not examine the association between gender (socially determined roles) and substance use or the association between the sex of youth with substance use conditions and the treatment they receive. Finally, this study did not examine family structure or the sex of the parent/caregiver who used substances to see if the pattern is similar (i.e., the male to female ratio of parents/caregivers who use substances).

## 6. Implications for Research and Practice

There are some important opportunities for future research to build upon from this study’s findings. Given the cross-sectional nature of this study, there is a need to develop prospective cohort studies to examine how early childhood factors and experiences may relate to the initiation or continuation of substance use, particularly following first contact with health services. The broad use of the interRAI ChYMH in several jurisdictions will afford opportunities to identify these cohorts and examine changes and outcomes over time in relation to substance use. Further information about the dose and frequency of substance use will enhance the rigor of these longitudinal studies, such as using the interRAI Addictions Supplement to the ChYMH instrument. Qualitative studies will complement these longitudinal studies to explore with youth, and their families or caregivers, reasons for substance use and factors that they feel could be leveraged to prevent future substance use.

There are several opportunities for future research to address limitations in the sex and gender analysis of substance use in this study. Considering this study’s patient type and setting (mental health), patterns of substance use by sex may have been subject to other factors not measured in this study (e.g., types of drugs). For instance, this study did not examine the association between gender (socially determined roles) and substance use or the association between the sex of youth with substance use conditions and the treatment they receive. Finally, this study did not examine family structure or the sex of the parent/caregiver who uses substances to see if the pattern is similar (i.e., the male to female ratio of parents/caregivers who use substances).

The different pattern of substance use among youth in the community and residential settings shows that care providers could intensify their efforts to identify and treat substance use conditions (initiation or disorder) among youth with less severe symptoms at first contact in all settings. Furthermore, based on the differences identified in the residential and community settings, treatments in the community setting could focus more on early identification, intervention, and monitoring, while the focus of treatment in the residential setting may be more about in-patient treatment and harm reduction. Also, comprehensive mental health assessment, including substance use assessment, should be completed for all youths seen in outpatient or residential settings at first contact. Substance use should also be considered within mental health treatment. Failure to treat mental health conditions that co-occur with substance use conditions and vice versa due to fragmented services would increase the burden on youth seeking treatment, leading to intensive treatment needs and premature termination, resulting in poor outcomes.

## 7. Conclusions

Individual and parental factors increase youth’s risk of substance use, highlighting the importance of a holistic approach to prevention/risk reduction, risk assessment, management, and recovery. There is no single cause of problematic substance use among youth. This study shows that exposure to traumatic life events, history of self-injurious ideation, parental substance use, and negative school experiences are strong predictors of substance use among youth. In contrast, a strong and supportive relationship with family is a protective factor. These results show that psychopathology and social factors (especially family and school) are extremely important in preventing and treating substance use among youth. Therefore, the prevention of substance use will require collaboration among many sectors of society. A comprehensive approach may focus on access to social and mental health supports, supportive education, reduced availability, and marketing restrictions.

## Figures and Tables

**Table 1:** Description of independent variables available from the interRAI ChYMH or ChYMH Screener.

Categories of Independent Variables	Biopsychosocial Model	Variables
Demographic information	Biological/genetic	Age, gender
Psychosocial needs	Psychosocial	residential instability, family functioning, social support, education engagement
Environmental factors	Environmental	Home environment
Clinical characteristics	Psychological, cognitive, personality	Mental health symptoms, cognition, harm to self, behavior, history of trauma or stress
Interventions		Treatment, control intervention, adherence to treatment, prevention
Service use history		Community mental health agency, residential/in-patient, emergency room

**Table 2:** Characteristics of the study population.

		Community						Residential					
		Total (N = 47,418)		M (N = 18,790)		F (N = 28,628)		Total (N = 700)		M (N = 377)		F (N = 323)	
Characteristics	Level	n	%	n	%	n	%	n	%	n	%	n	%
Instrument	ChYMH-S	38,972	82.2					4	0.6				
	ChYMH	8446	17.8					694	99.4				
Sex	Male	18,790	39.6					377	53.9				
	Female	28,628	60.4					323	46.1				
Age group	12–14	13,623	28.7	6350	33.8	7273	25.4	178	25.4	107	28.4	71	22
	15–16	18,149	38.3	6782	36.1	11,367	39.7	302	43.1	158	41.9	144	44.6
	17–18	15,646	33	5658	30.1	9988	34.9	220	31.4	11	29.7	108	33.4
Risk of disrupted education	Yes	27,580	58.6	11,859	63.6	15,721	55.2	417	61.6	236	65	181	57.6
	No	18,934	40.2	6503	34.9	12,431	43.7	238	35.2	111	30.6	127	40.5
	Not applicable	582	1.2	277	1.5	305	1.1	22	3.2	16	4.4	6	1.9
No Strong and supportive relationship with family	Yes	8542	18	2958	15.7	5584	19.5	238	34	107	28.4	131	40.6
	No	38,876	82	15,832	84.3	23,044	80.5	462	66	270	71.6	192	59.4
Parent/caregiver has mental health issue	Yes	17,150	36.2	6423	34.2	10,727	37.5	349	49.9	175	46.4	174	53.9
	No	30,268	63.8	12,367	65.8	17,901	62.5	351	50.1	202	53.6	149	46.1
Parental addiction/substance use	Yes	7537	20.1	3531	18.8	6006	21	279	39.9	153	40.6	126	39
	No	37,881	79.9	15,259	81.2	22,622	79	421	60.1	224	59.4	197	61
	≥5	1719	3.6	681	3.6	1038	3.6	43	6.1	23	6.1	20	6.2
Use of any substance in the last 14–30 days	Yes	10,587	22.3	4150	22.1	6437	22.5	259	37	139	36.9	120	37.1
	No	36,831	77.7	14,640	77.9	22,191	77.5	441	63	238	63.1	203	62.9
Behavior symptom	Yes	26,014	54.9	11,821	62.9	14,193	49.6	579	82.7	332	88.1	247	76.5
	No	21,404	45.1	6969	37.1	14,435	50.4	121	17.3	45	11.9	76	23.5
Self-injurious ideation or attempt	Yes	27,605	58.2	8217	43.7	19,388	67.7	458	65.4	201	53.3	257	79.6
	No	19,813	41.8	10,573	56.3	9240	32.3	242	34.6	176	46.7	66	20.4
Problematic sexual behavior	Yes	2683	5.7	954	5.1	1729	6	74	10.6	39	10.3	35	10.8
	No	44,735	94.3	17,836	94.9	26,899	94	626	89.4	338	89.7	288	89.2
Victim of abuse	Yes	17,247	36.4	5505	29.3	11,742	41	413	59	208	55.2	205	63.5
	No	30,171	73.6	13,285	70.7	16,886	59	287	41	169	44.8	118	36.5
Distraction and hyperactivity scale	0-None	9550	20.1	3056	16.3	6494	22.7	82	11.7	34	9	48	14.9
	1-Low	28,063	59.1	10,692	56.9	17,371	60.7	403	57.6	207	54.9	196	60.7
	2-Moderate	3719	7.8	1685	9	2034	7.1	61	8.7	34	9	27	8.4
	3-High	3023	6.4	1570	8.4	1453	5.1	78	11.4	56	14.9	22	6.8
	4-Very high	3063	6.5	1787	9.5	1276	4.4	76	10.9	46	12.2	30	9.3
Anxiety scale	0-None	8145	17.2	4484	23.9	3661	12.8	160	22.9	99	26.3	61	18.9
	1-Low	13,262	28	5751	30.6	7511	26.2	191	27.3	109	28.9	82	25.4
	2-Moderate	16,275	34.3	5844	31.1	10,431	36.4	204	29.1	107	28.4	97	30
	3-High	7839	16.5	2269	12.1	5570	19.4	105	15	54	14.3	51	15.8
	4-Very high	1897	4	442	2.3	1455	5.1	40	5.7	8	2.1	32	9.9
Depression severity Index	0-None	1921	4.1	993	5.3	928	3.2	40	5.7	24	6.4	16	5
	1-Low	20,186	42.6	9362	49.8	10,824	37.8	304	43.4	191	50.7	113	35
	2-Moderate	12,675	26.7	4828	25.7	7847	27.4	176	25.1	89	23.6	87	26.9
	3-High	5356	11.3	1708	9.1	3648	12.7	67	9.6	33	8.7	34	10.5
	4-Very high	7280	15.3	1899	10.1	5381	18.8	113	16.2	40	10.6	73	22.6
Sleep problem	Yes	29,868	63	10,853	57.8	19,015	66.4	415	59.3	222	58.9	193	59.8
	No	17,550	37	7937	42.2	9613	33.6	285	40.7	115	41.1	130	40.2
Cognitive problem	Yes	8254	17.4	4429	23.6	3825	13.4	326	46.6	207	54.9	119	36.8
	No	39,164	82.6	14,361	76.4	24,803	86.6	374	53.4	170	45.1	204	63.2
Last contact with CMH agency in the last year	No contact	14,973	31.6	6600	35.1	8373	29.2	91	13.2	47	12.7	44	13.7
	≥31	10,897	23	4435	23.6	6462	22.6	153	22.1	85	23	68	21.2
	≤30	21,547	45.4	7754	41.3	13,793	48.2	447	64.7	238	64.3	209	65.1

**Table 3:** The proportion of youth using substances in community settings, by gender.

		Total Community Sample				Males				Females			
Characteristics	Level	n	%	X^2^	*p*-Value	n	%	X^2^	*p*-Value	n	%	X^2^	*p*-Value
Sex	Male	4150	22.1	1.04	0.308								
	Female	6437	22.5										
Age group	12–14	683	5.0	4255.86	<0.0001	263	4.1	2205.8	<0.0001	420	5.8	2111.85	<0.0001
	15–16	4141	22.8			1652	24.4			2489	21.9		
	17–18	5763	36.8			2235	39.5			3528	35.3		
Contact with CMH	No contact in the last year	2823	18.9	172.06	<0.0001	1400	21.2	9.2	0.01	1423	17	218.37	<0.0001
	31+ days	2450	22.5			953	21.5			1497	23.2		
	30 days or less	5314	24.7			1797	23.2			3517	25.5		
Distraction and hyperactivity scale	0-None	1305	13.7	597.13	<0.0001	462	15.1	117.97	<0.0001	843	13	546.98	<0.0001
	1-Low	6614	23.6			2487	23.3			4127	23.8		
	2-Moderate	1104	29.7			445	26.4			659	32.4		
	3-High	805	26.6			379	24.1			426	29.3		
	4-Very high	759	24.8			377	21.1			382	29.9		
Anxiety Scale	0-None	1753	21.5	134.17	<0.0001	1052	23.5	10.56	0.032	701	19.2	187.13	<0.0001
	1-Low	2707	20.4			1243	21.6			1464	19.5		
	2-Moderate	3573	22.0			1255	21.5			2318	22.2		
	3-High	1991	25.4			487	21.5			1504	27		
	4-Very high	563	29.7			113	25.6			450	30.9		
Depression severity Index	0-None	317	16.5	758.76	<0.0001	188	18.9	161.48	<0.0001	129	13.9	637.69	<0.0001
	1-Low	3623	18.0			1831	19.6			1792	16.6		
	2-Moderate	2852	22.5			1064	22			1788	22.8		
	3-High	1422	26.6			483	28.3			939	25.7		
	4-Very high	2373	32.6			584	30.8			1789	33.3		
Sleep problem	No	3300	18.8	199.47	<0.0001	1527	19.2	64.73	<0.0001	1773	18.4	135.61	<0.0001
	Yes	7287	24.4			2623	24.2			4664	24.5		
Self-injurious ideation or attempt	No	2865	14.5	1214.5	<0.0001	1868	17.7	274.31	<0.0001	997	10.8	1070.65	<0.0001
	Yes	7722	28.0			2282	27.8			5440	28.1		
Behavior symptom	No	3690	17.2	614.59	<0.0001	1250	17.9	110.85	<0.0001	2440	16.9	520.45	<0.0001
	Yes	6897	26.5			2900	24.5			3997	28.2		
Problematic sexual behavior	No	7547	21.3	442.98	<0.0001	3892	21.8	14.36	0.0002	5655	21	546.12	<0.0001
	Yes	1040	38.8			258	27			782	45.2		
Cognitive problem	No	9299	23.7	260.42	<0.0001	3530	24.6	220.27	<0.0001	5769	23.3	63.86	<0.0001
	Yes	1288	15.6			620	14			668	17.5		
Victim of abuse	No	5073	16.8	1453.67	<0.0001	2499	18.8	282.72	<0.0001	2574	15.2	1238.69	<0.0001
	Yes	5514	32.0			1651	30			3863	32.9		
No Strong family relationship	No	7577	19.5	1001.43	<0.0001	3067	19.4	430.49	<0.0001	4510	19.6	575.47	<0.0001
	Yes	3010	35.2			1083	36.6			1927	34.5		
Parental addiction/substance abuse	No	7207	19.0	1183.86	<0.0001	2933	19.2	387.26	<0.0001	4274	18.9	798.18	<0.0001
	Yes	3380	35.4			1217	34.5			2163	36		
Parent with MH issue	No	6253	20.7	134.29	<0.0001	2655	21.5	8.02	0.005	3598	20.1		
	Yes	4334	25.3			1495	23.3			2839	26.5		
Risk of disrupted education	No	2795	14.8	1042.04	<0.0001	991	15.2	270.16	<0.0001	1804	14.5	805.99	<0.0001
	Yes	7545	27.4			3038	25.6			4507	28.7		
	NA	168	28.9			79	28.5			89	29.2		

**Table 4:** Pattern of substance use among youth with mental health conditions assessed in residential settings stratified by sex.

		Total Residential Sample				Male (N = 377)				Female (N = 323)			
		n	%	X^2^	*p*-Value	n	%	X^2^	*p*-Value	n	%	X^2^	*p*-Value
Sex	Male	139	36.9	0.006	0.94								
	Female	120	37.2										
Age group	12–14	29	16.3	48.82	<0.0001	20	18.7	24.1	<0.0001	9	12.7	25.39	<0.0001
	15–16	121	40.1			63	39.9			58	40.3		
	17–18	109	49.6			56	50			53	49.1		
Contact with Community Mental Health in Prior Year	No contact	31	34.1	2.09	0.35	24	51.1	5.06	0.08	7	15.9	11.21	0.004
	31+ days	51	33.3			27	31.8			24	35.3		
	30 days or less	175	39.2			86	36.1			89	42.6		
Distraction and hyperactivity scale	0-None	30	36.6	3.15	0.53	13	38.2	6.35	0.18	17	35.4	5.75	0.22
	1-Low	152	37.7			86	41.6			66	33.7		
	2-Moderate	27	44.3			12	35.3			15	55.6		
	3-High	27	34.6			17	30.4			10	45.5		
	4-Very high	23	30.3			11	23.9			12	40		
Anxiety Scale	0-None	77	48.1	11.83	0.02	56	56.6	23	0.0001	21	34.4	4.07	0.4
	1-Low	61	31.9			33	30.3			28	34.2		
	2-Moderate	69	33.8			33	30.8			36	37.1		
	3-High	39	37.1			14	25.9			25	49		
	4-Very high	13	32.5			*				10	31.3		
Depression severity Index	0-None	16	40	1.15	0.89	11	45.8	5.02	0.29	5	31.3	4.52	0.34
	1-Low	111	36.5			76	39.8			35	31		
	2-Moderate	65	36.9			26	29.2			39	44.8		
	3-High	28	41.8			14	42.4			14	41.2		
	4-Very high	39	34.5			12	30			27	37		
Sleep problem	No	95	33.3	2.77	0.1	55	35.5	0.2	0.64	40	30.8	3.8	0.05
	Yes	164	39.5			84	37.8			80	41.5		
Self-injurious ideation or attempt	No	64	26.5	17.67	<0.0001	58	33	2.17	0.14	6	9.1	27.97	<0.0001
	Yes	195	42.6			81	40.3			114	44.4		
Behaviour symptom	No	32	26.5	6.99	0.008	15	33.3	0.27	0.6	17	22.4	9.3	0.002
	Yes	227	39.2			124	37.4			103	41.7		
Problematic sexual behaviour	No	231	36.9	0.03	0.88	130	38.5	3.56	0.06	101	35.1	4.93	0.03
	Yes	28	37.8			9	23.1			19	54.3		
Cognitive problem	No	170	45.5	24.63	<0.0001	85	50	22.93	<0.0001	85	41.7	4.83	0.028
	Yes	89	27.3			54	26.1			35	29.4		
Victim of abuse	No	68	23.7	36.95	<0.0001	43	25.4	17.18	<0.0001	25	21.1	20.3	<0.0001
	Yes	191	46.3			96	46.15			95	46.3		
No Strong family relationship	No	147	31.8	15.65	<0.0001	87	32.2	8.83	0.003	60	31.3	7.06	0.008
	Yes	112	47.1			52	48.6			60	45.8		
Parental addiction/substance abuse	No	122	29	29.16	<0.0001	62	27.7	20.03	<0.0001	60	30.5	9.69	0.002
	Yes	137	49.1			77	50.3			60	47.6		
Parent with MH issue	No	130	37	0	0.98	76	37.6	0.11	0.745	54	36.2	0.1	0.75
	Yes	129	37			63	36			66	37.9		
Risk of disrupted education	No	55	23.1	28.18	<0.0001	22	19.8	18.68	<0.0001	33	26	10.5	0.005
	Yes	180	43.2			101	42.8			79	43.7		
	NA	11	50			8	50			*	*		

**Table 5:** Multivariable logistic regression using GEE examining factors associated with substance use among the community and residential settings by gender.

Multivariable Regression Analysis (GEE Model)				
Community			Residential	
Total Sample Model	Male Model	Female Model	Total Sample Model	
Variables	RR (95% CI)	RR (95% CI)	RR (95% CI)	RR (95% CI)
Sex (Ref = M)	0.88 (0.28, 0.94)			
Age Category				
12–14 (Reference)				
15–16	4.19 (3.61, 4.85)	5.63 (4.81, 6.59)	3.28 (2.81, 3.84)	2.11 (1.56, 2.85)
17–18	6.50 (5.53, 7.64)	8.96 (7.65, 10.5)	5.03 (4.23, 5.99)	2.52 (1.73, 3.65)
Risk of disrupted education:				
None (Reference)				
Yes	1.46 (1.35, 1.57)	1.39 (1.24, 1.56)	1.48 (1.39, 1.57)	1.89 (1.37, 2.61)
Not Applicable	1.35 (1.15, 1.60)	1.31 (1.07, 1.61)	1.35 (1.11, 1.64)	1.88 (1.22, 2.90)
Hyperactivity and Distractibility Scale				
None (Ref)				
Low	1.38 (1.26, 1.50)	1.37 (1.23, 1.51)	1.35 (1.23, 1.49)	
Moderate	1.58 (1.40, 1.79)	1.61 (1.39, 1.87)	1.53 (1.35, 1.74)	
High	1.57 (1.39, 1.77)	1.64 (1.43, 1.88)	1.49 (1.30, 1.70)	
Very high	1.59 (1.42, 1.78)	1.64 (1.42, 1.90)	1.55 (1.34, 1.78)	
Anxiety: None (Ref)				
ANX: Low	0.82 (0.78, 0.88)	0.83 (0.78, 0.89)	0.85 (0.79, 0.92)	0.60 (0.45, 0.80)
ANX: Moderate	0.75 (0.69, 0.82)	0.75 (0.69, 0.83)	0.78 (0.71, 0.86)	0.62 (0.48, 0.79)
ANX: High	0.71 (0.65, 0.77)	0.65 (0.57, 0.73)	0.76 (0.70, 0.83)	0.74 (0.51, 1.06)
ANX: Very high	0.69 (0.63, 0.75)	0.64 (0.53, 0.76)	0.73 (0.66, 0.80)	0.62 (0.38, 1.0)
Self injurious ideation or attempt	1.46 (1.34, 1.60)	1.25 (1.16, 1.36)	1.75 (1.56, 1.97)	1.55 (1.17, 2.06)
Behaviour symptom	1.36 (1.27, 1.44)	1.39 (1.27, 1.52)	1.31 (1.23, 1.39)	
Problematic sexual behaviour	1.39 (1.32, 1.46)	1.13 (1.02, 1.24)	1.51 (1.44, 1.58)	
Cognitive problem	0.70 (0.64, 0.77)	0.65 (0.58, 0.72)	0.77 (0.70, 0.85)	0.66 (0.52, 0.84)
Victim of abuse	1.27 (1.20, 1.34)	1.16 (1.09, 1.23)	1.34 (1.26, 1.43)	1.46 (1.19, 1.78)
No Strong family relationship	1.24 (1.17, 1.30)	1.31 (1.22, 1.41)	1.20 (1.13, 1.27)	
Parental addiction/substance abuse	1.45 (1.38, 1.52)	1.48 (1.37, 1.61)	1.40 (1.32, 1.47)	1.35 (1.11, 1.65)
Parent with MH issue			1.07 (1.02, 1.23)	

## Data Availability

Restrictions apply to the availability of these data. Like other ethics proposals using interRAI data (e.g., ORE 30173), data will be retained indefinitely in a secure location that is the interRAI Canada data server at the University of Waterloo. As part of the licensing agreement within interRAI, all instruments are provided to agencies, organizations and facilities free of charge. In return for the use of the instruments, data from each agency, organization de-identified data is given to interRAI to improve instruments, develop quality indicators, case mix system sand evidence-informed care planning initiatives to improve the care of vulnerable populations. However, for this study, investigators would only have access to the data for the duration of this project.

## Appendix A

**Table A1:** Characteristics of ChYMH and ChYMH-S Samples.

		ChYMH-Screener (N = 38976)		ChYMH (N = 9142)	
Characteristics	**Level**	**Freq**	**%**	**Freq**	**%**
Patient type	Community	38,972	99.99	8446	92.4
	Residential	4	0.01	696	7.6
Sex	Male	15,099	38.4	4068	44.5
	Female	23,877	61.3	5074	55.5
Age group	12–14	11,022	28.3	2779	30.4
	15–16	14,941	38.3	3510	38.4
	17–18	13,013	33.4	2853	31.2
Risk of disrupted education	Yes	23,280	60	4717	52.7
	No	15,145	39	4027	45
	Not applicable	392	1	212	2.4
No Strong and supportive relationship with family	Yes	6727	17.3	2053	22.5
	No	32,249	82.7	7089	77.5
Parent/caregiver has mental health issue	Yes	13,536	34.7	3963	43.3
	No	25,440	65.3	5179	56.7
Parental addiction/substance use	Yes	7691	19.7	2125	23.2
	No	31,285	80.3	7017	76.8
Highest number of alcohol drinks in the last (14–30 days)	None	34,723	89.1	8059	88.2
	1	1092	2.8	277	3
	2–4	1729	4.4	476	5.2
	≥5	1432	3.7	330	3.6
Intentional misuse of prescription or OTC medication	Yes	1197	3.1	502	5.5
	No	37,779	96.9	8640	94.5
Use of illicit drugs (in the last 14–30 days)	Yes	6568	16.9	1711	18.7
	No	32,408	83.1	7431	81.3
Use of any substance in the last 14–30 days	Yes	8583	22	2263	24.8
	No	30,393	78	6879	75.2
Behaviour symptom	Yes	20,654	53	5939	65
	No	18,322	47	3203	35
Self-injurious ideation or attempt	Yes	22,548	57.9	5515	60.3
	No	16,428	42.1	367	39.8
Problematic sexual behaviour	Yes	2332	6	425	4.7
	No	36,644	94	8717	95.3
Victim of abuse	Yes	13,875	35.6	3785	41.4
	No	25,101	64.4	5357	58.6
Distraction and hyperactivity scale	0-None	8061	20.7	1571	17.2
	1-Low	23,262	59.7	5204	56.9
	2-Moderate	3010	7.7	770	8.4
	3-High	2329	6	772	8.4
	4-Very high	2314	6	825	9
Anxiety scale	0-None	6729	17.3	1576	17.2
	1-Low	11,138	28.6	2315	25.3
	2-Moderate	13,498	34.6	2981	32.6
	3-High	6178	15.9	1766	19.3
	4-Very high	1433	3.7	504	5.5
Depression severity Index	0-None	1467	3.8	494	5.4
	1-Low	16,739	43	3751	41
	2-Moderate	10,490	26.9	2361	25.8
	3-High	4374	11.2	1049	11.5
	4-Very high	5906	15.1	1487	16.3
Sleep problem	Yes	24,797	63.6	5486	60
	No	14,179	36.4	3656	40
Cognitive problem	Yes	5908	15.2	2672	29.2
	No	33,068	84.8	6470	70.8
Last contact with CMH agency in the last year	No contact	13,301	34.1	1763	19.3
	≥31	8634	22.1	2416	26.5
	≤30	17,041	43.7	4953	54.2
